# Ethical issues in participatory arts methods for young people with adverse childhood experiences

**DOI:** 10.1111/hex.13314

**Published:** 2021-07-27

**Authors:** Gabriela Pavarini, Lindsay M. Smith, Nicola Shaughnessy, Anna Mankee‐Williams, Josita Kavitha Thirumalai, Natalie Russell, Kamaldeep Bhui

**Affiliations:** ^1^ Department of Psychiatry University of Oxford Oxford UK; ^2^ Wellcome Centre for Ethics and Humanities, Oxford Big Data Institute University of Oxford Oxford UK; ^3^ Department of Psychology, Institute of Psychiatry Psychology & Neuroscience King's College London London UK; ^4^ School of Arts University of Kent Canterbury UK; ^5^ Research and Innovation Department Falmouth University Falmouth UK; ^6^ St Anthony's Girls' Catholic Academy Sunderland UK; ^7^ Cornwall Council Cornwall UK; ^8^ Nuffield Department of Primary Care Health Sciences University of Oxford Oxford UK; ^9^ World Psychiatric Association UK Collaborating Centre Oxford UK; ^10^ Centre for Understanding Personality (CUSP) East London NHS Foundation London UK

**Keywords:** adverse childhood experiences, arts‐based methods, coproduction, ethics, mental health, neurodivergence, participation, participatory research, trauma, young people

## Abstract

**Context:**

Participatory arts‐based methods such as photovoice, drama and music have increasingly been used to engage young people who are exposed to psychosocial risks. These methods have the potential to empower youth and provide them with an accessible and welcoming environment to express and manage difficult feelings and experiences. These effects are, however, dependent on the way these methods are implemented and how potential ethical concerns are handled.

**Objective:**

Using the current literature on arts‐based health research as a foundation, this paper examines ethical issues emerging from participatory arts methods with young people with traumatic experiences.

**Results:**

We present a typology covering relevant issues such as power, accessibility, communication, trust and ownership, across the domains of partnership working, project entry, participation and dissemination. Drawing on our extensive clinical and research experiences, existing research and novel in‐practice examples, we offer guidance for ethical dilemmas that might arise at different phases of research.

**Conclusion:**

Adequate anticipation and consideration of ethical issues, together with the involvement of young people, will help ensure that arts methods are implemented in research and practice with young people in a fair, meaningful and empowering way.

**Patient or Public Contribution:**

The issues reviewed are largely based on the authors' experience conducting participatory research. Each of the projects referenced has its own systems for PPI including, variously, consultations with advisory groups, coproduction, youth ambassadors and mentor schemes. One of the coauthors, Josita Kavitha Thirumalai, is a young person trained in peer support and has provided extensive input across all stages.

## 1 ETHICAL ISSUES IN PARTICIPATORY ARTS METHODS FOR YOUNG PEOPLE WITH ADVERSE CHILDHOOD EXPERIENCES

### 1.1 The promise of arts‐based methods for young people with adverse childhood experiences

Adverse childhood experiences (ACEs) are potentially traumatic events that can have negative lasting effects on health and well‐being.^1^ This includes direct experiences of neglect; physical, mental or sexual abuse; adverse household experiences such as violence or substance abuse; and community risks such as poverty and peer victimisation. These experiences can have negative effects that extend into adolescence and beyond, including various mental health issues as well as behavioural and learning problems.^2^, ^3^ Researchers have documented challenges in verbal and emotional expression, attention, concentration and memory,^4^, ^5^ which together can create serious barriers to interventions that rely on verbal communication alone.

A promising way to overcome such barriers is through participatory arts‐based approaches, including improvisational theatre, music, dance, visual and digital arts activities. There is a long tradition of creative therapies and an expanding evidence base for applied, socially engaged and coproduced arts supporting mental health and well‐being.^6^ Creative practices offer a tool kit to explore and to express mental health experiences through methods that can safely transpose distressing thoughts and feelings into other modes or meaningful and symbolic representations.^7^, ^8^ Arts practices can be very diverse. This, however, can be a strength of the arts‐based approach, as the fact that different disciplines invite various levels and types of participation makes engagement more probable from a group itself likely to be diverse.

Creative practices work with techniques such as externalisation, metaphor, objects, puppetry, photography and group abstraction and use imagination to create distance from personal experience.^9^ Creative vocabularies are a means of articulating emotions and aspects of self that may be difficult to express and can contribute, for example, to internalising or externalising disorders in adolescence.^10^, ^11^ Hence, arts practitioners are increasingly engaging with participants' personal stories in ways that interact with therapeutic processes and which require specialist training to practice ethically, often in the context of Higher Education programmes in, for example, applied theatre, music or dance.

As many forms of participatory arts work include movement and embodied engagement, creative practices may also be helpful in managing the sensory effects of trauma, such as hyperarousal and somatic sensitivity, by supporting sensory integration and emotion regulation.^12^ Similarly, practices such as writing, drawing or improvisation can stimulate or support the imagination and allow ‘different’ stories to be explored. Interactive and group creative methods may additionally facilitate the development of coping skills and resilience to manage the potential psychosocial effects of trauma, including heightened experience of loneliness and sensitivity to potential social threats, perceived criticism and invalidation.^13^, ^14^ Arts‐based approaches overall may help children and adolescents identify environments and tools to build hope, confidence and social support as their engagement with a project unfolds. For example, simple but effective activities such as selecting beads that represent supportive others or personal strengths to later be strung onto a bracelet can provide adolescents with a continued reminder of internal and external support sources.^15^ Working within creative frameworks that are imagined and with objects, roles or metaphors provides a forum within which aspects of lived experience can be expressed and played with through encounters that develop agency. Participatory arts offer opportunities to take risks, discovering brave space through engagement in preference to avoidance.

Across diverse art forms and means of implementation, increasing evidence suggests that arts‐based methods, as embodied practices, support young people's agency and empowerment and allow them to express fully their experiences and challenges.^6^, ^16^, ^17^, ^18^ Creative methods may be better for reaching marginalised young people, for example, those from minority backgrounds (e.g., gender and sexual minorities, neurodivergent youth and ethnic minorities) who are known to experience barriers to more conventional forms of engagement or mental health support,^19^, ^20^, ^21^ and they experience particular traumas more frequently or differently.^22^, ^23^, ^24^, ^25^, ^26^, ^27^ These and other groups may find in arts‐based projects an accessible and less stigmatising way of expressing, documenting and processing emotional experiences.

The therapeutic success of creative methods within care settings has inspired an interest in the use of arts‐based methods in practice and in participatory research.^28^, ^29^, ^30^ Participatory or emancipatory research values a commitment to creating spaces for children and adolescent voices to be fully heard and prioritised in the research process.^31^ Rather than taking on a passive role as subjects of research, adolescents are seen as activists and actors who set the agenda for research; coproduce interventions; collect and generate data; analyse results; and disseminate outputs. Art, in its broadest sense, can be used as a medium through which to coproduce research questions, generate data, interpret or perform data or disseminate findings; art products may constitute research objects or dissemination outputs in their own right,^29^ creating curiosity and connections between art‐makers, and diverse observers and communities.

Alongside the increased attention to participatory community research in terms of cocreation, lived experience and authenticity, the 21st century has witnessed a questioning of the relationship between performance and participation.^32^ This has involved calls for a more nuanced language, attention to different forms of evidence and an intersectional understanding of participation and agency that is attentive to the role played by environments, social relations and subjectivities.^33^ This paper deliberately keeps the definition of ‘the arts’ broad, as the practices best suited to different needs are also likely to be broad and diverse. Art forms such as creative writing, music, theatre and visual arts offer a range of opportunities for engagement and therapeutic benefit that can be tailored to individuals and/or groups through the cocreation process.

Ensuring arts‐based health research (ABHR) is empowering, inclusive and transformative for youth depends on the way these methods are implemented. Indeed, several unique ethical considerations emerge when working at the research intersection between arts practice and personal experiences related to trauma. There is a growing evidence base in the broader field of ABHR that has attempted to taxonomize ethical issues using case studies as a foundation.^28^, ^34^, ^35^, ^36^ Yet, there remain calls for greater scrutiny of ethical issues in ABHR and the development of theory and practical guidelines.^37^, ^38^, ^39^, ^40^ In what follows, we aim to contribute to this evidence base by examining issues emerging from work with adolescents and, in particular, in the field of trauma or ACEs.

## 2 ETHICAL CONSIDERATIONS IN ARTS‐HEALTH RESEARCH WITH ADOLESCENTS WITH ACEs

Our analysis considers ethical issues that emerge throughout the lifetime of ABHR with adolescents, from creating partnerships, working together, through to dissemination. We provide creative solutions to address ethical issues, largely emerging from the authors' work with participatory arts research projects involving individuals who have experienced or who are vulnerable to ACEs. These are ongoing in the UK and include ‘Playing A/Part’, ‘Imagining Autism’, ‘Imagining Futures’, ‘Theatre Troupe’ and ‘HeadStart Kernow’. To protect the anonymity of project participants in relation to the issues discussed, we refrain from citing issues as related to particular projects. For further information on the scope of the projects, please see the acknowledgements section. A summary of core ethical issues and creative solutions is provided in Table 1. While our discussion is broadly relevant, the examples and relevant legislation that we cite are from the United Kingdom, where we have conducted most of our research and intervention work. When applying our typology to their own work, researchers should also be guided by relevant cultural expectations and local regulatory standards.

**Table 1:** Ethical issues and creative solutions in participatory arts methods for young people with adverse childhood experiences

Phase of project	Ethical issues	Creative solutions
Partnership working	▪ Power: Imbalance of power within research/partner relationships (funding, organisational and societal status). ▪ Regulations and infrastructure: Questions as to the appropriateness of the role of REC boards in defining and adjudicating on ethical issues related to interdisciplinary practice. ▪ Expertise: Differential expertise, training and understanding in working with young people with ACEs. ▪ Expectations: Differential expectations in terms of timeframes, experience, biases, values outputs and codes of practice.	▪ Conscious challenge and flattening of pre‐existing hierarchy through frontloaded dialogue and structured cultural exchange within a design thinking co‐lab environment, rooted in empathy and young person (user)‐centred. ▪ All partners receive training in how to ‘hear’ rather than ‘listen’. ▪ Cocreating a code of conduct, set of values and language for the environment and communications. ▪ Knowledge and skill set analysis for all project partners including roles, responsibilities and contributions at the outset of the project. Negotiation on expectations and aims. ▪ Clear identification and fulfilment of training needs for project partners (formal and informal).
Project entry	▪ Reach and accessibility: Enabling breadth and depth of participant reach by transdisciplinary arts/health study design identifying and addressing stigma, anonymity, confidentiality, equality issues and the intersecting spaces between these elements that challenge meaningful reach and accessibility through arts‐based approaches. ▪ Consent: Ensuring informed consent can be provided by children and young people for participation in the intervention; participation in the research; and the collection and sharing of data. ▪ Legislation: Facilitating the delivery of multilayered organisational and legislative requirements for participation, intervention, sharing of data and creation and curation of art outputs. Clarity of obligations for confidentiality versus information sharing with parents/carers and wider systems.	▪ Within a design thinking colab environment, cocreate study design with young people that also mitigates the potential pressurising effect of ‘study design’ and outcomes focus on artistic experience as a priority and facilitates reach and accessibility for participants. ▪ Proactive consultation with parents (where appropriate), and other services and youth organisations to seek guidance, support and shared decision making regarding safeguarding. ▪ Consideration of the appropriateness of risk screening methods depending on the project and the participants. ▪ Cocreate with children and young people; informed consent documentation and guidance documents for all aspects of the project that is accessible, age appropriate, with supporting contemporary communication channels with messaging cocreated and codelivered with young people. ▪ Clear, accurate and accessible communications about the scope of the project including arts and health aims and processes.
Participation	▪ Communication: Awareness, value, methods and respect of the multiplicity of communication including verbal and nonverbal language, cultural norms, experiences, translation and interpretation and channels. ▪ Trust: Meaningful participation driven by genuine creative enquiry that may raise perceived levels of risk to/for participants and therefore drive a more limited ‘statutory requirement’ approach to activity and ethics. ▪ Distress: Potential for retraumatization in exploring own experiences and those of others in the group. ▪ Monitoring and support: Challenges of monitoring the impact of participation (mental and physical health). (1) Outside of the participant activity and schedule e.g., flashbacks or rumination, (2) supporting disclosures and meaning making from ACEs. ▪ Subjectivity: Issues of ‘truth’, honouring participant interpretation and representation in any artistic abstraction with varying levels of sensitivity to art products, visual, auditory and immersive.	▪ Developing creative confidence by cocreating approaches that: ▪ Respect and embed the Rights of the Child. ▪ Enable equality and embrace diversity. ▪ Address implicit bias. ▪ Respect pronouns and identity. ▪ Implement communication badge approach (e.g., sign language). ▪ Embed a ‘traffic light’ system for feelings and risk. ▪ Enable participation pivot if the environment changes (e.g., COVID‐19). ▪ Create a safe/sensory‐sensitive space. ▪ Develop and embed a code of practice/group agreements on behaviours. ▪ Use of ‘time‐out’ areas. ▪ Provide appropriate support in the event of escalation of need. ▪ Discuss signs and symptoms of distress and identify with young people acceptable communication methods within and between practice sessions including feedback mechanisms. ▪ Use of mentors and peer support initiatives to monitor and manage risk. ▪ Between‐workshop communication and consultation with parents and service supports.
Dissemination	▪ Authorship and ownership: Rights of acknowledgement versus protection of anonymity. ▪ Public domain exposure: Potential for stigmatisation through surfacing of personal narratives expressed through art, with the potential of audience/viewer misinterpretation or inadvertent harm to audience. ▪ Cultural differences: Interdisciplinarity difference in understanding and cultures of arts‐based data analysis, quality, aesthetics and value, publication, collaboration and notions of ethical research. Potential for exploitation of participants and their artwork for others' gains.	▪ Recurrent discussion with participants about rights and desires for acknowledgement in various outputs, including consideration of potential later regrets if pieces of work are/are not personally identified in relation to the project. Right to forget or change narrative. ▪ Explore consent for potential dissemination/discussion of work as part of pseudo‐anonymized vignettes and agree parameters for use of work and personal information in vignettes. ▪ Establish individual rights for use of participants' own artwork for their own purposes. ▪ Within a design thinking colab environment throughout the lifetime of the project cocreate the dissemination methodology with all partners and young people rooted in empathy and young people (user)‐centred, whilst considering audience impact and social responsibilities. ▪ Engagement with international arts‐based health researchers and practitioners on best practice in developing project‐specific frameworks regarding rights and ownership, safeguarding and communication.

### 2.1 Partnership working

Each of the practice disciplines that might contribute within the field of ABHR is underpinned by its own professional code of ethics and conduct, with shared central values guiding practice, including respect (for autonomy), competence, responsibility, integrity, openness, honesty and beneficence.^41^, ^42^, ^43^, ^44^ Yet, previous studies have drawn attention to ethical issues emerging at the very outset of projects, in partnership working and the bringing together of practitioners from different disciplines.^36^

The very language of the varied disciplines working at the intersection of arts and health research may differ, affecting interpersonal perceptions and relational dynamics.^45^, ^46^ Developing a shared use of language is particularly important when considering the appropriateness of terms of reference when working with ‘marginalized’ or ‘vulnerable’ groups and intergenerationally, in conversation and broader communications. There is great potential to stigmatise, offend or disenfranchise young people participating in ABHR, as a result of esoteric or inaccessible language or overmedicalization. Experience of ACEs and trauma are prevalent amongst minority groups, including neurodivergent young people, lesbian, gay, bisexual and queer (LGBTQ) youth and ethnic minorities,^23^, ^47^ and there may exist additional language issues to discuss with project participants, including for example, the use of preferred pronouns and identity first language.^48^, ^49^

Practitioners from different disciplines, with different expertise, also necessarily relate to and comprehend the personal experience of ACEs and mental health issues in different ways.^50^ When working with individuals who have experienced trauma, this may lead to tensions in the role of researchers and practitioners, in balancing approaches of engagement, taking therapeutic or positive risks, empowerment and freedom of individual creative practice.^36^, ^51^ There may be requirements to deliver specific additional training up front, over and above basic safeguarding, for example, in trauma‐informed care, and to share differing areas of expertise and approaches. Previous studies have used specific ‘risk of harm’ protocols and have highlighted the importance of outlining specific roles and close collaboration between project staff, parents and local health services for signposting.^52^ It is also necessary to consider provisions and supports for staff well‐being in this context^53^, ^54^ and to include, for example, times for debriefing and reflective practice.

Differences in the role and place in society within which practitioners from arts and health research disciplines conceive of themselves, and their relationship to hegemonic structures, can also pose significant ethical tensions right at the heart of partnership working.^36^, ^55^, ^56^ Power imbalances in partner participation may emerge as a result of discrepant funding or status in academic and clinical organisations and arts/community partners.^36^ Shortcomings have been highlighted by previous studies in applying institutional Research Ethics Committee mechanisms to community and participatory projects, including ideas that knowledge generation rests with the clinical researchers.^57^, ^58^

Matarasso questions in ‘A Restless Art’^59^ ‘whose interests are being served by a participatory arts project’ and ‘who defines the aims’ and what is valued in the process or outcome? Significant prior negotiation and planning is required to address core issues of power and purpose, and to resolve potential tensions in, for example, academic obligations of truth and accuracy versus arts abstraction, addressing what is valued in academic projects and managing expectations regarding requirements and how to measure impact and dissemination.^56^, ^60^

Most acutely, when working with young people who may have significant negative experiences of exclusion, loss of agency or control, there is a need to work on means by which to ‘flatten the hierarchies’ in operational structures. For example, in the work of the Playing On Theatre Company, collaborative practices engage clinicians, service users and arts practitioners in theatre‐making activities, where the identities and roles of those involved are not revealed at the start of the process.^61^

A seeming majority of studies grappling with ethical issues in ABHR understandably identify codesign and coworking with young people as key to addressing these issues creatively and respectfully. However, implementing an involvement strategy for any research project raises its own ethical issues about balancing meaningful involvement with other project delivery pressures.^62^, ^63^ Whilst many projects may acceptably elect for the inclusion of potential participants in consultative involvement roles, ‘co‐design’ suggests that benefits in terms of engagement and project efficacy can be delivered by authentic and equitable collaboration between stakeholders.^64^ This can be hard to achieve in practice and there remain opportunities, for example, in researching young people's reflections on overcoming ethical issues in ABHR.^65^

### 2.2 Project entry

It is often claimed that arts‐based methods provide a familiar and more accessible medium through which to communicate with or ‘reach out’ to adolescents who have had experiences of trauma and those from marginalised groups and communities.^66^, ^67^ Arts activities that do not solely prioritise high levels of literacy may be more appealing and accessible to young people who are more likely to have experienced challenges at school, poor attainment and school exclusion as a result of ACEs.^68^, ^69^ The buildings themselves that are used as community arts spaces may be geographically more accessible or better designed for diversity, and less likely to attract the stigma that may be associated with statutory mental health or health research facilities.^70^, ^71^ However, when aiming to promote inclusion, particularly for young people and individuals with experiences of social adversity and exclusion, there remain several potential barriers to participation in ABHR and ethical issues to consider at project entry.^62^

It is not a given that arts activities are more accessible for young people with trauma, and ‘the arts’ can be perceived as elitist and exclusionary if care is not taken in the presentation and communication about projects.^72^ Arts‐based research projects that exist to support young people with ACEs will likely be aiming to reach diverse participants, from more marginalised communities. There is an ethical obligation, in line with the Equality Act 2010,^73^ to attempt to maximise the opportunities and means by which people might be invited into projects, to ensure representation. This will depend on forging close links with representative organisations from education, faith or cultural communities, youth sector organisations and local branches of charitable and third sector supports for LGBTQ and neurodivergent youth, for example. Inclusion also depends on the provision of appropriate compensation for participation, as well as subsistence and transport when applicable.^74^ There is a wide range of options when compensating adolescents, from direct employment to providing vouchers or certificates. It is essential that young people value what is provided and consider it a fair return to their contribution.

Another key ethical concern at project entry for ABHR concerns communication with young people about the aims and research components, and protecting the rights of children to express and receive information under the United Nations Convention on the Rights of the Child.^75^ The association with ACEs, trauma, mental health or diversity is potentially stigmatising, and whilst it is clear that the research aims must be explained to young people in accessible and inclusive language (visually and with aids if needed), there remains debate as to the detail, depth and language that is used to describe study/project aims. For example, to what extent are expected change processes made explicit with young people at project outset? In ABHR, there is a balance to be struck between ensuring accurate information about the research elements of a project to support informed choice, whilst not undermining the accessibility that an arts focus might deliver for some young people. Coproducing recruitment posters, information sheets, consent forms and other entry‐point materials with the target audience can help improve access to the project and participants' understanding of the research.

In addition to enhancing the quality of the information provided to participants, it is also important that young people participate in setting the research goals and outcomes and engage in critical reflection upon the context in which constructs are defined. In one of our projects, for instance, autistic female participants questioned whether standardised well‐being measures captured their lived experiences. These reflections are key to producing ABHR that responds to the priorities and experiences of the groups involved, and that is sensitive to differences between the populations being evaluated. A lack of resonance might prevent adolescents from engaging in the first place.

Indeed, several previous studies have drawn attention to issues of consent and assent in ABHR with young people.^35^, ^37^, ^52^, ^58^, ^76^ The consenting process is a legally binding agreement made with someone who has the capacity to consent. Gaining assent is a process of assessing the wishes of a child (without full capacity) in relation to research, promoting understanding and gaining an affirmative agreement to participate. Consent to participate is not something that may be sought just at project outset in ABHR, but rather, ongoing and recurrent processes of consent and assent are often required as regards participation in the arts activities themselves and the sharing of any arts outputs. There may also be phases or elements of the research that are distressing, or trigger distress, and young people should be entitled to review their participation or receive support around decision making. External influences and life events may also impinge on their desire or willingness to remain involved.

Specific consents may be required for different aspects of the project, including research participation, participation in an intervention, data sharing with different parties and photography and recording processes.^77^, ^78^ In the United Kingdom, there is no clear statute governing children's right to consent to take part in research, except from clinical trials of investigational medicinal product,^79^ but consent from parents or legal guardians is typically required for those under 16 years and advised for those under 18 years.^80^, ^81^, ^82^

Opt‐out consent is often used in educational and community settings, whereby a child's consent is obtained, and parents are given the chance to object to their child's inclusion within a reasonable timeframe.^83^, ^84^ Obtaining valid consent from a child requires an assessment of whether participants can be considered ‘Gillick competent’.^79^, ^82^ ‘Gillick competence’ is a term used in medical law to decide if a child under 16 years old is able to consent to his or her own treatment, without the need for parental permission. The application of Gillick competence to research requires consideration of whether a minor is able to understand the nature and outcomes of the project and his or her rights as a participant. In such studies, the potential influence of power dynamics between the researcher, gatekeepers and youth must be carefully assessed.^83^, ^85^

Even when adolescents are deemed competent, it is still good practice to involve parents or guardians in the decision‐making process.^82^, ^86^ Parents play an important role in assessing information about research studies and supporting an adolescent's decision about participation. They can also offer guidance and a reassuring presence during the research.^87^ It is important to note, however, that relationships with parents may be strained or absent for young people with past trauma or ACEs,^88^ and there is evidence that requiring active parental consent may limit the participation of adolescents with self‐reported adverse outcomes.^89^ Parental involvement can limit participation of LGBTQ+ youth in gender and sexual health studies, particularly those who hold negative self‐views or lack family support,^90^, ^91^, ^92^ and the participation of youth in digital mental health intervention research.^93^

These emerging findings pose an urgent need for empirical ethics research with adolescents and parents to better understand potential implications of guardian permission requirements and to design consent strategies that protect minors without silencing high‐risk participants. Researchers and ethics committees need to consider carefully the risks and benefits of participation; the necessity, feasibility and impact of different types of consent processes for the target audience; and young people's vulnerability, agency and competency.^94^, ^95^ In online ABHR, additional potential barriers to obtaining consent must be considered, such as challenges around age identification.^96^, ^97^

Care must also be taken to ensure that the desire to benefit from a project's arts activities does not result in a young person forging a particular identity around specific experiences or mental health labels to which they might not have done in other circumstances, and potentially to their detriment. The Children's Acts 1989 and 2004^98^, ^99^ remind us that the welfare of the young person is paramount. There exist specific welfare issues regarding the timing of inviting young people's participation in arts‐based projects related to ACEs or trauma. As Gubrium et al.^51^ describe, there is a fine line between protecting participants with trauma history from further harm and patronising them through social exclusion. Screening methods may be used helpfully to ascertain risk and safeguarding issues at project entry, but potentially at the expense of accessibility.^52^ Working together with the young person, alongside their advocates, including parents and other support services, is vital to the process of assessing the suitability of ABHR for any particular young person.^94^, ^100^

### 2.3 Participation

Paramount to ABHR is the creation of accessible and appealing spaces for participation.^101^ The potential for retraumatizing inadvertently in the environment or set‐up of a space or activity is something that needs careful consideration *before* the start of the project, particularly for young people who may have just started to recover from ACEs.^51^ A variety of tools have been developed, with safety and ethics in mind, to support creative practitioners to make decisions during workshops or rehearsal processes where participants' personal or collective stories might be used. For example, ‘the Drama Spiral method’ in participatory theatre^102^ provides a way to assess and regulate the degree of ‘emotional distance’ that any games, exercises or creative activities have from a person's life story. The Spiral is a diagram comprised of six concentric rings on which a facilitator (or participant) can plot activities, ranging from games or fictional narratives, to personal and sensitive stories, as one spirals towards the centre. Pragmatic guidance is given on contracting with people as to the remit of any activity and work, identifying aims and establishing boundaries, for example. Somewhat similarly, the ‘Risk Table’ developed in training and projects with young refugees and asylum seekers allows practitioners to map artistic practices against the level of personal (emotional and psychological) and creative risk in each activity and approach.^19^ Each planned activity can be plotted on a diagram with level of personal risk and artistic risk as *X*–*Y* axes. Emphasis is placed on building towards more creative risk slowly, through consultation with young people, and driven by them. There is no obligation to take personal risks within the creative practices, although individuals may be supported to do so, by practitioners who are skilled in supporting the process, always guided by the principles of choice, respect and equality. These methods have been developed to minimise the risk of retraumatizing young people, by placing emphasis on activities such as arts making, rather than therapy, and by distancing from potential reactivation of traumatic memories.

Young people have varying levels of sensitivity to art products, including visual, auditory or kinaesthetic. The sensory needs and differences of neurodivergent individuals are also important factors in considering accessibility, safety and agency in mixed groups. Potential adverse effects must be considered in the context of these sensitivities and the stage that adolescents with ACEs have reached in their identity formation and recovery process. Ongoing monitoring of potential adverse effects is also crucial, both during and after sessions (e.g., flashbacks or rumination). Young people often arrive at participatory arts projects having experienced continued disempowerment, exclusion and scepticism of their capacity by adults or other youth in their lives. Meeting children's right to a voice, as stated in the United Nations Convention on the Rights of the Child,^75^ means more than providing space—it requires taking a number of steps to foster adolescents' sense of agency and ability to participate meaningfully.^103^

Respecting and valuing different ways of communicating, language and experiences is crucial to creating a positive environment. Creating a culturally sensitive environment might include a series of adjustments and adaptations such as offering interpreters to facilitate communication, or selecting meeting venues that feel accessible and familiar to different cultural groups. It might also include training researchers and facilitators to improve cultural competencies regarding the needs of particular groups. For instance, specific training on the health needs of lesbian, gay, bisexual, trans and intersex people can promote awareness of inequalities as well as inclusive and nonjudgmental communication and care.^104^

Researchers must also take into consideration young people's own beliefs and experiences in relation to mental health and support services, and potential social and political barriers to their expression of agency. Understanding and valuing participants' realities and knowledge systems is essential to foster an atmosphere of belonging, trust and safety.^105^ The same applies to participants' interpretation and representation of any artistic abstraction: honouring participants' ‘truth’ is a key aspect of ethical practice in participatory arts.^106^ Finally, fostering critical awareness of one's own identity and mutual understanding of motivations and values is also essential to deconstructing pre‐existing biases and promoting an environment of greater emotional understanding and empathy.^107^

What *safety* means also differs for different individuals. It is essential that adolescent participants take an active role in defining the group's code of practice or agreement according to their own needs and priorities.^108^, ^109^ This collective process should take place at the outset of any project and iterated as new issues might arise throughout the lifetime of the project. In one of our projects, for instance, participants coproduced a poster with the code of practice, which included items such as ‘everyone has the right to speak or pass’ and ‘all questions are good questions’. We displayed the poster at the meeting venue throughout the project; any participant was welcome to add or edit items as the project unfolded. The ability to choose and shape the topics of discussion and activities also supports adolescents' ability to express their views. It is also worth noting, however, that while speech is important to agency, participants must not feel pressured to express their views. Not speaking or nonparticipation also needs respecting and valuing.

Indeed, the relations between voice and speech are complex and there is a need to reconsider how voice can move beyond speech. Working in group contexts to create collective stories can also be a means of finding expression for experiences that are difficult for individuals to articulate. Having this witnessed can be crucial to effecting change for those involved. Attention must also be paid to youth preferences for communication channels, both within and in between participatory arts sessions. With regard to digital meetings, the opportunity to make contributions using the chat function within a videoconference or via notes on joint online boards such as *Padlets* might facilitate the participation of young people who might not be willing to speak up in a group setting. When choosing virtual platforms to host meetings and communicate remotely, researchers must consider the balance between accessibility/engagement and data privacy/safety. Platforms that are highly accessible to youth might not offer acceptable risk level or comply with relevant local legislation such as the UK Data Protection Act^110^ if personal and/or confidential information is shared.

It is equally important to monitor and address participants' well‐being as activities unfold. Clear knowledge of what to do and who to approach when someone is uncomfortable or experiencing distress is critical. There are a number of creative solutions in attempting to improve the perceived ‘safety’ of any environment. One such solution consists of creating a ‘sensory space’ or restorative niche^111^—a place that individuals can access anytime during the session when they feel the need to. The use of communication badges that indicate how participants feel each day can also serve as helpful cues to guide how participants communicate with one another and the facilitator. Some researchers adopt a traffic light system, whereby participants indicate varying levels of vulnerability, risk or communication preferences. These can change as appropriate to the context, timing or tasks. In addition to having a counsellor available, training some of the participants to act as the first point of contact for safeguarding concerns can also be a helpful solution, given that adolescents often look to their peers for support.^112^ Cocreating internal codes to facilitate a welcoming environment is also important. In one of our projects, for example, participants agreed on specific Zoom icons to express support whenever someone disclosed something personal and potentially vulnerable.

The structure of each session will be highly dependent on the nature of the project and the needs of adolescent participants. In our experience, participants often find it helpful to follow a general session structure, balanced with open‐ended activities that afford better freedom and space to be creative. Collective agenda setting can also facilitate involvement and ownership over the research process.^108^ Similarly, giving adolescent participants control over the pace and sequence of activities, and letting individuals and groups adapt activities to their own needs can be helpful. Such flexibility is an important aspect of safeguarding, along with providing support and guidance and monitoring participants' needs. In our own work with adolescents, deeply emotional narratives often arise spontaneously, as peer interactions unfold during participatory research sessions. Facilitators must be prepared to handle such episodes and flexibly adapt the sessions according to the group's current needs.

Last but not the least, we find it important to emphasise that *risk* is not necessarily negative. Taking risks can be important to learning, self‐development and to change. In participatory arts, risk‐taking is acknowledged as a ‘core principle’^113^ and the ethics associated with this are integral to the field of research, which emphasises the importance of playfulness to meaning making and experiential learning, the concept of ‘critical vulnerability’ and the necessity for safe structures in which risks can be taken. Useful distinctions have been made between creative and personal risk, with the former being facilitated and supported through strategies that minimise the latter.^19^ Striking the right balance between growth and vulnerability is essential to good practice in participatory arts research.

### 2.4 Dissemination

Ethical review of health‐related research projects is traditionally guided by the principles of maintaining participant anonymity and confidentiality.^114^ Protocols for anonymizing and storing participant data, as well as explaining the boundaries of confidentiality to young people, will form a central part of the procedural ethics review for any study.^82^, ^94^ However, arts‐based projects are typically based on a process of creating something that is then shared with others, or creating together as a shared process. Acknowledging and communicating one's contributions to this process may be a part of delivering the potential benefits of artistic approaches in trauma work. This includes changes to self‐concept; increased confidence; the development of new meanings about experiences; sense of connection with others; and reduced stigma.^115^, ^116^ Just as practitioners must respect the artist's right to anonymity, they must also respect the right to the acknowledgement of any individual's creative process. However, when working with young people with trauma histories, there is a need to explore sensitively not only thoughts and feelings in relation to certain artistic outputs now but also how they might potentially feel about this expression being in the public in the future, and the possibility of later regrets or changes to one's narrative.^51^ These intricacies require specific protocols that take into account the context and needs of each particular project. Careful consideration is needed to decide on the appropriate scale or audience for the dissemination of any work.^117^ Certain artistic outputs might be best kept within a small sharing, with a closed group. However, if dissemination is to involve a wider audience, for example, in a performance piece or gallery showing, then several additional ethical issues emerge.

In projects where identity disclosure is judged appropriate, additional challenges might arise in obtaining appropriate consents for acknowledgement where more than one individual is represented in the work.^118^ The potential impact of dissemination on any audience must also be considered.^117^ It is commonplace now to see ‘trigger warnings’ on performance pieces, and yet, there is ongoing debate as to whether these are helpful or harmful to individuals.^119^ Individual artists are permitted to share their own subjective artistic expressions as they please. However, the responsibilities of ABHR projects may not be equitable. Where an audience may include family members and siblings of participants, there is a need to consider the potential for inadvertent harm.^117^ There will always be potential for viewer misinterpretation in any shared piece of work,^120^ but arguably, ABHR projects may have a greater responsibility not to misrepresent certain health issues or glorify or dramaticize certain negative aspects of experience. There is also the question of whether and how much to disclose about the research element of the project to any audience.^121^ Equally, criticisms have been raised regarding the exploitation of the arts or under‐representing of the artistic contributions within the dissemination of health research findings via traditional publication routes.^122^

ABHR projects also raise critical ethical issues about the ownership of new learnings and findings, and particularly of the artworks themselves.^28^ This includes what licence individual participants, facilitating artists, project leads and researchers have over the use and sharing of information or artwork derived from any project.^40^, ^123^ There may be a requirement for new legal and contractual structures to support interdisciplinary research collaboration that acknowledges the rights of contributing artists and participants, over and above service or organisation‐level contracts. Sharing of information online, via social media and various software platforms creates additional issues and potential needs for safeguarding in dissemination. Whilst it is a simpler process to ensure that participants refrain from documenting or sharing experiences or artworks during the participation in workshops, via group privacy agreements and social media guidelines, the use of artworks and outputs following completion of a project is less regulated.

## 3 CONCLUSION

We have considered ethical issues in ABHR involving adolescents who have experienced or who are vulnerable to ACEs. There has been much recent interest and suggested potential in the role of arts‐based methods in managing and recovering from trauma, and yet, research scrutiny and examination of process are comparatively lacking. Based on the literature and our own work with participatory arts projects, we have presented creative solutions to ethical challenges in this area for review and ongoing commentary. Work in the field of trauma raises specific issues regarding the potential for adverse secondary effects and requires careful consideration as a result of the immense scope and diversity of personal experience and recovery.

Our viewpoint has been necessarily broad, as research at the intersection of arts practices and trauma is still emerging. We aimed to demonstrate that review of ethical issues must be an ongoing process, embedded within ABHR from partnership building to dissemination. The process of continued ethical analysis and cocreation of solutions must involve participants and any target audience, to ensure appropriate and effective solutions.

Best practice guidance will necessarily differ depending on the population targeted and research themes. Similarly, the salience of different ethical issues will vary depending on the artform (e.g., literary, visual or performative) and whether activities take place online or in person. Our understanding of how arts‐based participatory methods can empower and transform the lives of adolescents with ACEs will be greatly enhanced by future ethics research with increased focus on the specific arts methods and mechanisms and their relationship to specific cognitive experiences of trauma and developmental processes in adolescence.

## ATTUNE COLLABORATIVE

This manuscript was written on behalf of the ATTUNE Collaborative, which comprises: University of Oxford: Prof. Kamaldeep Bhui, Prof. Mina Fazel, Dr. Gabriela Pavarini Falmouth University: Prof., Eunice Ma, Ms. Anna Mankee Williams, Prof. Tanya Krzywinska, Prof. David Prior. University of Kent: Prof. Nicola Shaughnessy Leeds University: Prof. Siobhan Hugh‐Jones Queen Mary University of London: Dr. Sania Shakoor, Dr. Georgina Hosang, Dr. Bridgette Escolme, Dr. Michaela Otis King's College London: Prof. Craig Morgan, Dr. Lindsay Smith University College London: Prof. Peter Fonagy, Prof. Daisy Fancourt National Society for the Prevention of Cruelty to Children: Pam Miller Young People Cornwall: Nick Smith Cornwall Council and HeadStart Kernow: Charlotte Hill, Natalie Russell Centre for Mental Health: Andy Bell Cornwall Partnership NHS Trust: Prof. Rohit Shankar, Mrs. Sharon Hudson.

## CONFLICT OF INTERESTS

The authors declare that there are no conflict of interests.

## AUTHOR CONTRIBUTIONS

Gabriela Pavarini and Lindsay M. Smith wrote the first draft of the manuscript. All authors contributed to the typology, critically revised the manuscript for important intellectual content and approved the final version.

## Data Availability

Data sharing not applicable to this article as no datasets were generated or analysed during the current study.
